# The Role of MRI in Evaluating Spinal Cord Injuries: Diagnostic Accuracy, Prognostic Value, and Clinical Decision-Making

**DOI:** 10.7759/cureus.87040

**Published:** 2025-06-30

**Authors:** Khaled Agha Tabari, Shivling S Swami, Alousious Kasagga, Amanuel Kefyalew Assefa, Maysaa N Amin, Rahma Hashish, Ann Kashmer Yu

**Affiliations:** 1 Radiology, Queen Elizabeth University Hospital, Glasgow, GBR; 2 Internal Medicine, California Institute of Behavioral Neurosciences and Psychology, Fairfield, USA; 3 Pathology, Peking University, Beijing, CHN; 4 Orthopaedics and Trauma, University Hospitals of Leicester NHS Foundation Trust, Leicester, GBR; 5 Microbiology/Immunology, California Institute of Behavioral Neurosciences and Psychology, Fairfield, USA; 6 Internal Medicine, Sherwood Forest Hospitals NHS Foundation Trust, Nottingham, GBR

**Keywords:** acute spinal cord injury (asci), magnetic resonance imaging (mri), mri spine, role of mri, spinal cord injury

## Abstract

Spinal cord injury (SCI) happens when the spinal cord is damaged, which can be caused by different types of trauma or other non-traumatic factors. These injuries are a major contributor to disability and long-term morbidity on a global scale, often resulting in severe neurological deficits and complications. While radiography and computed tomography (CT) frequently provide guidance for initial assessment, magnetic resonance imaging (MRI) is the most effective method for imaging soft tissues. MRI is essential in identifying and managing SCI because it can demonstrate relevant details, detect bleeding, swelling, and ligament damage, and offer valuable insights for prognosis. This traditional review evaluates the role of MRI in managing SCI by examining relevant literature. The review examines MRI's diagnostic accuracy, its value in predicting outcomes, and how it influences clinical decisions. The paper provides a well-rounded view of its advantages and limitations. It includes full-text review articles and meta-analyses published in English between 1996 and 2024, with additional studies included if the selected articles reference them.

Our findings demonstrate that MRI is crucial for diagnosing acute SCI due to its ability to provide detailed visualization of spinal cord pathology, which other imaging techniques often miss. It aids in prognosis, with features like intramedullary hemorrhage and lesion length correlating with neurological recovery; however, features of edema and cord compression are not as clear-cut when it comes to predicting outcomes. MRI also influences surgical planning and timing through its ability to assist in determining the need for decompression and stabilization procedures. Future research should focus on developing standardized MRI criteria and conducting high-quality trials to enhance the clinical utility, prognostic reliability, and diagnostic accuracy in the treatment of SCI.

## Introduction and background

Spinal cord injury (SCI) happens as a result of trauma (such as falls, sports-related injuries, or traffic accidents) or non-traumatic causes (such as tumors, vascular and degenerative diseases, infections, poisons, or congenital disabilities) [1]. Any damage to the spinal cord falls into the category of an SCI [2]. Depending on whether there is any movement or feeling at or below the site of the damage, an injury is classified as complete or incomplete [3]. It can result in severe complications, including paraplegia, quadriplegia, and mortality [4]. The number of patients suffering from SCI has exceeded 15 million, with more than 4.5 million years of human life having been lived with spinal cord impairment [1]. The mean age at injury rose from 29 to 43 years between the 1970s and 2015 [5]. As of 2012, over 80% of reported injuries were among males [6]. Although typical lengths of stay at acute hospital units have decreased significantly since the 1970s, they have remained at a staggering 12 days since 2015. In 2023, individuals with paraplegia had an average yearly expense of over $650,000 in their first year [5]. Accurate and efficient use of MRI in evaluating SCI can help optimize care, hence reducing unnecessary interventions and healthcare costs. Those affected by SCI are more likely to develop severe and possibly life-threatening conditions, which can lead to premature mortality. To lessen the worldwide burden of SCI, effective prevention, treatment, rehabilitation, and continuing medical care are crucial [1].

Traumatic injuries are the cause of approximately 90% of SCIs [7]. Spinal injuries caused by trauma can lead to localized impairments, neurological abnormalities, and even potentially fatal circumstances [8]. The injury can have many consequences, from temporary discomfort to total paralysis. The American Spinal Injury Association (ASIA) Impairment Scale is used to classify the severity of spinal injuries based on sensory and motor elements. There are five grades within this classification [9].

**Figure 1:**
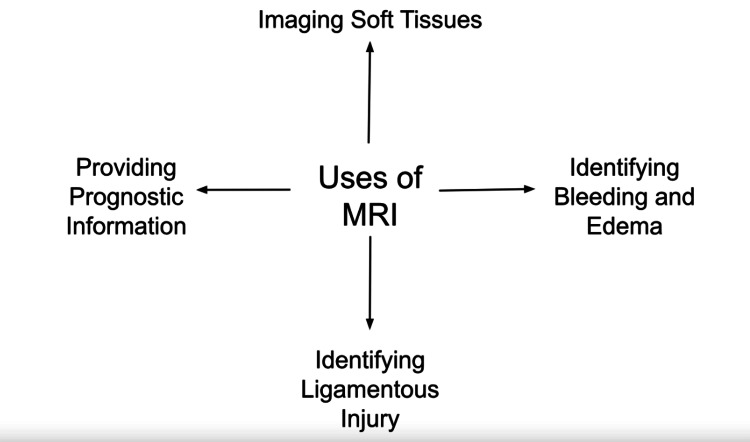
Uses of MRI Figure created by Dr. Khaled Agha Tabari using Google Docs.

Multiple imaging methods aid in the diagnosis of spine disorders. Radiography is the first-line imaging modality in patients without neurological deficits or symptoms in the thoracic or lumbosacral regions [10]. X-rays enable us to visualize the vertebral column, assess the status of the spinal canal, and identify changes such as osteoarthritis and ossification of the posterior longitudinal ligament. However, radiography has not been acknowledged as the most reliable method for the diagnostic workup of the spine because it is impossible to detail the spinal cord and nerve roots [11]. Computed tomography (CT) is quick, non-invasive, and precise [11] and is widely used for cervical trauma screening [12]. However, its ability to visualize soft tissues is poor [13]. Conversely, magnetic resonance imaging (MRI) is considered a vital diagnostic tool for SCIs due to its exceptional capacity to image soft tissues, identify bleeding, edema, and ligamentous injuries, and provide prognostic information [14,15], as illustrated in Figure 1.

Although MRI is widely used, it is associated with several issues and safety concerns. Firstly, it is contraindicated in patients with cardiac pacemakers and implanted metalwork. It is challenging to perform an MRI on claustrophobic patients since they sometimes cannot remain in the scanner for the complete evaluation. Moreover, MRI is generally contraindicated in pregnancy, particularly in the first trimester [11].

This traditional review aims to evaluate the function of MRI in its ability to accurately diagnose conditions, prognosis prediction capability, and impact on clinical outcomes. The approach will be to carefully analyze recent, relevant data on the role of MRI in assessing and managing SCI. The review aims to inform clinicians and highlight areas requiring further research.

## Review

MRI is an essential component in detecting disc, spinal cord, ligamentous, and soft-tissue injuries [16]. The MRI is considered the gold standard imaging technique in SCI [17] and offers the best contrast resolution. It plays a significant role in managing acute spinal injuries [14]. Given that we already have sufficient evidence highlighting the role of imaging modalities and the significance of SCIs, this section explores the diagnostic, prognostic, and clinical decision-making implications of MRI. This section further explores each of the stated domains while identifying research gaps and offering future recommendations.

Diagnostic accuracy

One of the studies reviewed assesses the importance of MRI in acute SCI [15]. The paper emphasizes its accuracy as a diagnostic tool compared to other imaging modalities, such as CT and X-ray. According to researchers [15,18], MRI is an excellent tool for identifying disc herniations, ligamentous injuries, spinal cord diseases, and epidural hematomas. The signals of fat, proteinaceous fluid, subacute hemorrhage, and intravascular contrast agents are all amplified in T1-weighted images. In contrast, edema fluid and bulk phase are hyperintense on T2-weighted imaging [19]. Moreover, MRI is crucial for assessing complicated trauma because it can evaluate brain and extraneural damage in various planes. It is a necessary technique in the management of acute SCI because it offers comprehensive, thorough insights into vascular diseases, such as artery dissections, and injuries like traumatic disc herniation (TDH) [15].

It is noted that MRI's superior contrast resolution and multiplanar imaging capabilities make it the gold standard for identifying spinal disorders [11]. It offers high-resolution viewing of soft tissues and bone, which allows us to accurately identify features of vertebral bodies, intervertebral discs, nerve roots, and the spinal cord [11]. T2-weighted imaging is most helpful in detecting spinal cord edema, inflammation, or central stenosis. T1-weighted imaging is excellent for visualizing bone marrow alterations and osseous structures [11]. Fat-suppression techniques, such as STIR (short tau inversion recovery), allow clinicians to identify minor lesions or bone marrow edema [11].

In trauma cases, MRI can detect ligamentous injuries, concealed fractures, and intraspinal hematomas. Furthermore, it can reveal diagnostic markers such as T2 signal alterations and gadolinium (Gd) enhancement for spinal infections. Additionally, MRI is crucial for diagnosing inflammatory conditions, including multiple sclerosis, malignancies, and degenerative diseases [11]. Notwithstanding its benefits, MRI has drawbacks. For instance, it is less sensitive to calcified structures than CT and is contraindicated for individuals with metal implants or pacemakers [11].

Prognostic value

Extensive research is available on the predictive significance of MRI in SCI, especially in SCIWORA (spinal cord injury without radiological abnormality). Researchers have highlighted the importance of MRI in assessing recovery outcomes in SCIWORA cases [20]. They found a strong relationship between MRI results and long-term prognosis. In particular, the evidence gathered highlights a negative correlation between MRI alterations and both recovery pace and final motor score, indicating that more widespread MRI changes in the spinal cord were linked to worse recovery prospects [21]. Moreover, a pronounced negative correlation was found between prevertebral hyperintensity length on MRI and ASIA Impairment Scale (AIS) scores at presentation and follow-up and recovery rates [21].

Interestingly, SCIWORA cases with spinal cord edema and normal MRI findings recovered more quickly, suggesting that MRI may be able to offer helpful prognostic information even in the absence of apparent structural damage [21]. On the other hand, when MRI results revealed cord contusion and swelling, recovery prospects were much lower [21]. These findings are in line with those of another prominent study [22]. The findings demonstrated that SCIWORA patients without detectable abnormalities had a more remarkable mean improvement in AIS grades than those with detectable abnormalities [22], thus highlighting the prognostic utility of MRI. Nevertheless, little information regarding the long-term prognosis is available after two years. Some patients may continue to improve, while others may have worsening circumstances due to recurrent injuries or abnormalities such as scoliosis [23]. These findings demonstrate the complexity of the prognosis for SCIWORA yet confirm the vital role that MRI plays in the initial assessment and the prediction of long-term recovery.

MRI plays a crucial role in prognostication following acute SCI, with several MRI features demonstrating an association with neurological recovery. One of the most significant predictors of poor outcomes is the presence of intramedullary hemorrhage, which we identify as a marker of more severe injury. According to a research article [24], three studies found that hemorrhage on MRI was predictive of worse neurologic recovery [24-27], while two others reported no association [28,29].

In some instances, it was noted that all patients with intra-axial hematoma had complete motor and sensory loss (Frankel grade A) and did not show neurological improvement at follow-up [27]. Additionally, longer rostrocaudal hemorrhage lesion lengths may occasionally lead to worse recovery [24,27,28]. The length of hemorrhage appears to correlate with prognosis; some findings suggest that each millimeter increase in hemorrhage length raises the risk of retaining a complete SCI, with patients with longer hematomas demonstrating no improvement in AIS grade [28]. Conversely, a hemorrhage length of less than 4 mm was associated with better neurological recovery [28]. Furthermore, one study revealed that cord compression from extra-axial hematomas was not associated with poorer recovery [27]. Table 1 summarizes key findings from various studies, illustrating the relationship between these MRI features and patient outcomes following SCI.

**Table 1:** MRI Features and Prognostic Implications for Neurological Recovery in Acute Spinal Cord Injury MRI: Magnetic Resonance Imaging; SCI: Spinal Cord Injury

MRI Feature	Study/Author(s)	Association With Recovery	Details/Findings
Intramedullary Hemorrhage	Kurpad et al. [24]	Predictive of worse neurological recovery	Three studies found hemorrhage on MRI linked to poor outcomes.
	Selden et al. [27]	Complete SCI (Frankel grade A) with no improvement	All patients with intra-axial hematoma had complete SCI and no recovery at follow-up.
Rostro-Caudal Hemorrhage Length	Kurpad et al. [24], Selden et al. [27], Boldin et al. [28]	Longer length linked to worse recovery	A hemorrhage length of more than four mm is associated with a poor prognosis. Boldin et al. [28] reported that each additional mm of hemorrhage length increased the risk of complete SCI.
Cord Compression From Extra-Axial Hematomas	Selden et al. [27]	There is no association with poorer recovery	Compression from extra-axial hematomas was not linked to worse neurological recovery.
Hematoma Length Under 4 mm	Boldin et al. [28]	Better neurological recovery	Hemorrhage length of less than 4 mm was associated with improved recovery.

Other MRI parameters, such as maximum spinal cord compression (MSCC) and maximal canal compromise (MCC), showed inconsistent associations with neurological recovery [25,27,30]. Some studies suggest that a lower MCC is predictive of worse outcomes, while others have found no significant correlation [24,25,30].

Several researchers have investigated the relationship between MRI findings and neurological recovery in individuals with SCI. Three studies found no direct link between cord edema (high T2 signal intensity) and neurologic outcomes [25,28,29], though two reported that longer edema lesion lengths predicted worse recovery [26,28]. We have encountered conflicting results regarding SCI lesion length and cord swelling, with some studies finding associations with poor prognosis, while others did not [27,30]. Additionally, single studies found no significant correlation between neurologic recovery and factors such as soft-tissue injury, preinjury stenosis, disc herniation, cord contusion, rostral edema location, or baseline sensory and motor scores [24].

Two articles examined the relationship between MRI findings and functional recovery in SCI [30,31]. Researchers assessed functional outcomes using the Functional Independence Measure (FIM), motor scores, functional dependence, and self-reported measures of manual dexterity and pain [24]. One study found that lower MCC was linked to worse FIM scores but did not impact manual dexterity or pain [24]. Meanwhile, MSCC and spinal canal diameter did not predict functional outcomes [30]. A longer SCI lesion length was associated with poorer manual dexterity and increased pain but showed no correlation with FIM scores [30]. Lastly, MRI findings of edema or hemorrhage were unreliable predictors of functional recovery [31].

Several research articles have explored the prognostic value of MRI in acute SCI, with varying findings. Research on SCIWORA suggests that MRI is crucial in predicting recovery, even in the absence of structural damage. Atesok et al. [20] found that more extensive MRI changes in the spinal cord, such as cord contusion and swelling, were associated with worse recovery. In contrast, SCIWORA cases with spinal cord edema but no structural abnormalities had better outcomes. In contrast, broader studies on SCI prognosis, such as Kurpad et al. [24], emphasize intramedullary hemorrhage as a key predictor of poor recovery, with longer hemorrhage length correlating with worse neurological outcomes. In contrast to Atesok et al., Kurpard et al. [24] highlighted that some studies found that edema lesion length and SCI lesion length predicted worse outcomes, while others reported no significant association. This contradiction highlights inconsistencies in MRI-based prognostication across these two studies.

Although Atesok et al. was published more recently, Kurpad et al., as a systematic review, follows a more rigorous methodology, minimizing bias through structured inclusion criteria and a broader scope. Unlike Atesok et al., which focuses solely on SCIWORA, Kurpad et al. examine all patients with acute SCIs, making their findings more generalizable and clinically applicable [24].

Functional outcomes, as assessed using measures such as the FIM, were not consistently linked to MRI findings, with factors like MSCC and MCC showing mixed results [24]. There is uncertainty regarding the ability of MRI to predict long-term recovery, as some patients may improve while others deteriorate due to secondary complications. These findings demonstrate that MRI is essential for predicting short-term prognosis; however, its accuracy varies significantly in long-term predictions, depending on the injury type and imaging findings.

Impact on clinical decision-making

One systematic review and meta-analysis emphasizes MRI's pivotal role in clinical decision-making for acute SCI. The findings show that MRI influenced surgical decisions in 36% of cases, guided the choice of surgical approach in 29%, and impacted the timing of surgery in 78% [13]. Furthermore, the findings suggest that MRI did indeed significantly impact clinical decisions. The study states that MRI helped determine whether instrumentation was needed and which spinal levels to decompress when indicated. Lastly, it also helped identify which patients required additional surgery. This study demonstrates the potential of MRI to improve patient care and its usefulness in identifying SCIs. The evidence supports MRI as an essential tool for preoperative planning in cases of acute SCI, although more prospective studies are required.

Moreover, other data indicate that MRI has a significant impact on clinical decision-making for SCI. Although it does not present large amounts of data, it highlights the importance of MRI in assessing spinal cord damage and describes it as an instrumental tool in determining the level of spinal cord compression. It is also worth noting that MRI helps inform surgical strategies. One of the studies cited by Kurpard et al. demonstrated that MRI results can determine the timing of surgical intervention and also offer initial management options [32]. Nevertheless, there are serious methodological flaws in this study, such as selection bias and poor-quality evidence [24]. To validate the deductions made, the authors emphasized the need for high-quality, well-designed studies to accurately determine the influence of MRI on decision-making and patient outcomes [24]. Another study further emphasized the lack of reliable data on the impact of MRI on clinical outcomes. The authors recommended the use of MRI with caution when making clinical judgments. However, the quality of the evidence provided was also low [24,33].

Due to its thorough synthesis of high-quality studies and rigorous methods, the systematic review and meta-analysis by Ghaffari-Rafi et al. [13] is more substantial when compared to other studies. Ultimately, it provides more reliable evidence of MRI's influence on clinical judgment. In contrast, the review by Kurpard et al. [24] has multiple limiting factors, including the small number of studies referenced, methodological flaws, and relatively older data.

Limitations

This review was limited to English free full-text review articles and meta-analyses published between 1996 and 2024. The databases used to gather and select articles were PubMed and Google Scholar. However, this review also references studies that did not meet these criteria, as the review articles and meta-analyses cited them. One of the limitations of this review article is that it did not account for the various MRI techniques, such as T1- and T2-weighted imaging or diffusion tensor imaging, used in different studies. The lack of control over variables such as injury type, patient population, age, and severity of the injury also reduces the credibility of the findings. There was also variability in the outcome measures in the chosen articles, for instance, the use of several clinical assessment scales and different follow-up durations.

Additionally, the data would have been more generalizable if high-quality randomized controlled trials (RCTs) were studied and if there had been a greater emphasis on chronic injuries. The variability in study quality and methodological detail among the selected articles may have introduced bias. Consequently, small sample sizes among some of the references and a lack of MRI standardization further limit the objectivity of this study.

Future research should ideally utilize more extensive and rigorous data, including standardized variables such as age, type of injury, and severity. Moreover, long-term patient follow-ups would occur over a substantial period (ideally, two years or more). Lastly, comparative studies may be beneficial as they offer insight into how well different MRI sequences predict neurological outcomes.

## Conclusions

In conclusion, this study explains why MRI is the gold standard imaging tool for SCI. This imaging tool provides us with detailed spinal imaging and precise diagnoses while also enabling the detection of subtle injuries. We can also deduce initial management plans and sometimes predict clinical outcomes through the use of MRI. Subsequently, more thorough research is required to reliably predict long-term prognosis and its impact on decision-making. Future research should focus on enhancing MRI techniques, particularly in the context of complex injuries. There should also be an emphasis on conducting large-scale studies that utilize standardized criteria. Lastly, it is important to address current limitations, such as contraindications and accessibility issues, to optimize its use in clinical settings.
